# Book Reviews

**Published:** 1902-02

**Authors:** 


### BOOK REVIEWS.

STUDIES IN THE PSYCHOLOGY OF SEX. SEXUAL INVERSION. By Havelock Ellis, L.S.A. (England); Fellow of the Medico-legal Society of New York and the Anthropological Society of Berlin; Honorary Fellow of the Chicago Academy of Medicine, etc.; general editor of the Contemporary Science series since 1899. The "Studies in the Pscychology of Sex" will probably be completed in five volumes. "Sexual Inversion" is second volume in the series. Pages xi-272. Size $8\frac{5}{8}$ x $5\frac{3}{4}$ inches. Extra cloth, $2.00 net, delivered. Sold only to physicians, lawyers, advanced teachers, and scientists. Philadelphia, Pa.: F. A. Davis Co., Publishers, 1914-16 Cherry Street.

This volume is concerned with homo sexuality in men and in women. Numerous cases are reported and views upon the subject cited *in extenso*. There are added chapters on homo sexuality in tramps, by Josiah Flynt, Ulrich's views, school friendships among girls and an account of the urning, Countess Sarolta V.

The book is an interesting study of a peculiar phase or deviation from the normal in human nature, and is a valuable addition to the literature of the subject.

---

THE MEDICAL NEWS POCKET FORMULARY, NEW (4TH) EDITION. Containing 1700 prescriptions representing the latest and most approved methods of administering remedial agents. By E. Quin Thornton, M.D., Demonstrator of Therapeutics, Pharmacy and Materia Medica in the Jefferson Medical College, Philadelphia. New (4th) edition, carefully revised to date of issue. In one wallet-shaped volume, strongly bound in leather, with pocket and pencil. Price, $1.50, net. Lea Brothers & Co., Philadelphia and New York, 1902.

The fourth edition of this formulary contains, in addition to the formulas of former editions, many combinations of the newer remedies. Among these and among the newer applications of older remedies may be mentioned the use of hydrogen dioxide in the removal of powder stains, urea in the treatment of lupus, apomorphia as a sedative in alcoholic delirium, guaiacol in promoting the absorption of serous exudates, chloresone and orthoform as local anesthetics, the substitution of the organic forms of silver and arsenic for the older and more poisonous inorganic salts. The book is handsomely bound and of convenient size for carrying around. It is fully indexed in every particular.

---

MANUAL OF PHYSICAL DIAGNOSIS. For the use of Students and Physicians. By James Tyson, M.D., Professor of Medicine in the University of Pennsylvania and Physician to the University Hospital; Physician to the Philadelphia Hospital; Fellow of the College of Physicians of Philadelphia; Member of the Association of American Physicians, etc. Fourth Edition, Revised and Enlarged. With Colored and other Illustrations. Published by P. Blakiston's Son & Co., 1012 Walnut Street, Philadelphia. 1901. 12mo. Cloth, $1.50 net.

The fourth edition of this work is but slightly larger than the preceding editions, but contains a number of new illustrations and some additions to the text. Besides the ordinary topics of physical diagnosis it contains chapters on the Roentgen Ray in diagnosis and on making an autopsy. The book is a clear and careful exposition of the subject, and has been for a long time well known and well liked as a smaller text-book.

---

LEA'S SERIES OF POCKET TEXT-BOOKS: HAYDEN ON VENEREAL DISEASES. A Pocket Text-book of Venereal Diseases. For Students and Practitioners. By James R. Hayden, M.D., Chief of Clinic and Instructor in Venereal and Genito-Urinary Diseases in the College of Physicians and Surgeons, New York, etc. New (3d) edition, thoroughly revised. In one handsome 12 mo volume of 304 pages, with 66 engravings. Cloth, $1.75 net. Flexible leather, $2.25 net. Lea Brothers & Co. Publishers, Philadelphia and New York.

This manual has already achieved popularity and the new edition will be welcomed. It contains in the way of new material sections on vegetations and herpes prepatiatis. The book is designed to give a practical knowledge of chancroid, gonorrhea, stricture and syphillis, and this it does in a brief, concise manner. It can be recommended as one of the best of its kind.

---

A BRIEF MANUAL OF PRESCRIPTION WRITING in Latin or English for the use of Physicians, Pharmacists, and Medical and Pharmacal Students. By M. L. Neff, A.M., M.D., Cedar Rapids, Ia. Pages v-152. Size, 8 x 5¾ inches. Extra cloth, 75 cents net, delivered. Philadelphia, Pa.: F. A. Davis & Co., Publishers, 1914-16 Cherry Street.

This small work is designed for the instruction of medical students and that very numerous class of physicians whose knowledge of the correct form of prescription writing leaves much to be desired. By both it may be very profitably read.

